# Evaluation of living liver donors using contrast enhanced multidetector CT – The radiologists impact on donor selection

**DOI:** 10.1186/1471-2342-12-21

**Published:** 2012-07-24

**Authors:** Kristina Imeen Ringe, Bastian Paul Ringe, Christian von Falck, Hoen-oh Shin, Thomas Becker, Eva-Doreen Pfister, Frank Wacker, Burckhardt Ringe

**Affiliations:** 1 Department of Diagnostic and Interventional Radiology, Hannover Medical School, Carl-Neuberg Str. 1, 30625, Hannover, Germany; 2 Department of General, Visceral and Transplantation Surgery, Hannover Medical School, Carl-Neuberg Str.1, 30625, Hannover, Germany; 3 Department of General and Thoracic Surgery, University Hospital Schleswig-Holstein, Arnold-Heller Str.1, 24105, Kiel, Germany; 4 Department of Pediatric Gastroenterology and Hepatology, Hannover Medical School, Carl-Neuberg Str. 1, 30625, Hannover, Germany; 5 Drexel University, College of Medicine, 216 N Broad Street, Philadelphia, PA, 19102, USA

**Keywords:** LDLT, Transplantation, Living donor, Recipient, CT

## Abstract

**Background:**

Living donor liver transplantation (LDLT) is a valuable and legitimate treatment for patients with end-stage liver disease. Computed tomography (CT) has proven to be an important tool in the process of donor evaluation. The purpose of this study was to evaluate the significance of CT in the donor selection process.

**Methods:**

Between May 1999 and October 2010 170 candidate donors underwent biphasic CT. We retrospectively reviewed the results of the CT and liver volumetry, and assessed reasons for rejection.

**Results:**

89 candidates underwent partial liver resection (52.4%). Based on the results of liver CT and volumetry 22 candidates were excluded as donors (31% of the cases). Reasons included fatty liver (n = 9), vascular anatomical variants (n = 4), incidental finding of hemangioma and focal nodular hyperplasia (n = 1) and small (n = 5) or large for size (n = 5) graft volume.

**Conclusion:**

CT based imaging of the liver in combination with dedicated software plays a key role in the process of evaluation of candidates for LDLT. It may account for up to 1/3 of the contraindications for LDLT.

## Background

Since the first report of successful living donor liver transplantation (LDLT) in 1990 [1], LDLT has become a valuable treatment for patients with end-stage liver disease who cannot receive deceased donor livers. Particularly in children who need a small sized graft, the use of liver transplantation is limited due to shortage of deceased donor organs.

Since the safety of volunteer living donors in LDLT has always been considered paramount, evaluation of potential candidates plays a crucial role to confirm suitability and to identify possible contraindications. Each transplant center has its own protocol for living donor evaluation, which typically includes a comprehensive medical and psychosocial examination as well as non-invasive imaging and other studies to assess size, anatomy and function of the liver.

Both, contrast enhanced computed-tomography (CT), and magnetic resonance imaging (MRI) have been shown to be suitable diagnostic tools, and are being used concurrently in different centers throughout the world. Imaging in a living liver donor has three objectives: (1) to identify any intraparenchymal lesions or abnormalities like fatty changes; (2) to visualize the extra- and intrahepatic vascular and biliary anatomy; and (3) to determine the size of the whole liver and calculate the graft and remnant liver volumes. The main advantage of CT over MRI is based on a higher spatial resolution and manifold post-processing possibilities [2,3].

The preoperative imaging process in LDLT is demanding and conscientious. Radiologists play a key role in filtering and providing the required information to surgeons. In addition, they may help to identify unsuitable donors and avoid unnecessary or invasive studies and procedures. The purpose of our study was thus to evaluate the significance of multidetector CT in this process, and therefore to reflect the radiologists impact on donor selection.

## Materials and methods

This retrospective study was approved by the ethics committee of Hannover Medical School with a waiver of consent granted.

### Candidate donors and recipients

Between May 1999 and October 2010 170 candidate donors (92 female, 78 male, mean age 39 years, range 18-61 years) underwent biphasic CT of the liver (Table 1). In addition, all donors had routine ultrasound examination. Further, biliary anatomy was assessed intraoperatively. The recipients were 143 patients (53 female, 90 male; 99 children, 44 adults) with a mean age of 15.3 years (4 months-71 years) (Table 2). In 121 recipients one donor was evaluated by means of CT, in 19 recipients two donors, in 1 recipient three donors, and in 2 recipients four donors, respectively. Most common underlying diseases were biliary atresia in children (n = 69), liver cirrhosis (n = 19), and hepatocellular carcinoma respectively hepatoblastoma (n = 22). We retrospectively reviewed the results of the CT and liver volumetry, and reasons for rejection were assessed.

**Table 1:** Candidate donor demographic data

**Number of potential donors**	**170**	
Donor age (years)	18-61 (mean 39)	
Donor sex (male / female)	78 / 92	
Performed LDLT	89	*Mean graft volume [ml]*
Left lateral	57	277 (SD 63)
Full left lobe	1	414
Right lobe	31	1134 (SD 317)

LDLT = living donor liver transplantation; SD = standard deviation.

**Table 2:** Recipient demographic data

**Number of potential recipients**	**143**
Patient age	4 months - 71 years (mean 15.3 years)
Patient sex (male / female)	90 / 53
Underlying disease	
Biliary atresia	69
Liver cirrhosis	19
Tumour (HCC, Hepatoblastoma)	22
PSC, PBC	7
α1-Antitrypsin deficiency	6
Alagille syndrome	4
Acute liver failure	2
Other	14

### Image acquisition

Until October 2005 CT was performed using a 4-channel multi-detector row CT (Somatom Plus 4A, Siemens, Erlangen, Germany). To keep radiation dose levels as low as possible, a native CT scan was not acquired. 150 ml of a nonionic iodinated contrast agent (Ultravist 300®, Bayer Schering Pharma, Berlin) followed by a 40 ml saline flush (NaCl 0.9%) were injected at a flow of 3-5 ml/sec. Biphasic image acquisition of the liver started 5 seconds after bolus detection in the abdominal aorta for the arterial phase. Portal-venous phase scanning followed after an interscan delay of 15 seconds. The parameters were identical for both scans: 3 mm slice collimation, a table feed of 5 mm per gantry rotation, and 2 mm reconstruction interval. Starting in October 2005 CT was performed using a 64-channel scanner (Lightspeed VCT, GE Medical Systems, USA). Multiphase scanning was performed after intravenous injection of 120 ml of a nonionic iodinated contrast medium (Imeron 400®, Nycomed GmbH, Germany) followed by a 50 ml saline flush (NaCl 0,9%) at a rate of 5 ml/sec. Arterial dominant phase images were obtained 15 seconds after bolus detection in the abdominal aorta, portal-venous scanning followed after an interscan delay of 25 seconds. Again, the parameters were identical for both scans: 1.25 mm slice collimation, a table feed of 39.37 mm per gantry rotation, and 1 mm reconstruction interval.

### Image analysis and postprocessing

**Figure 1:**
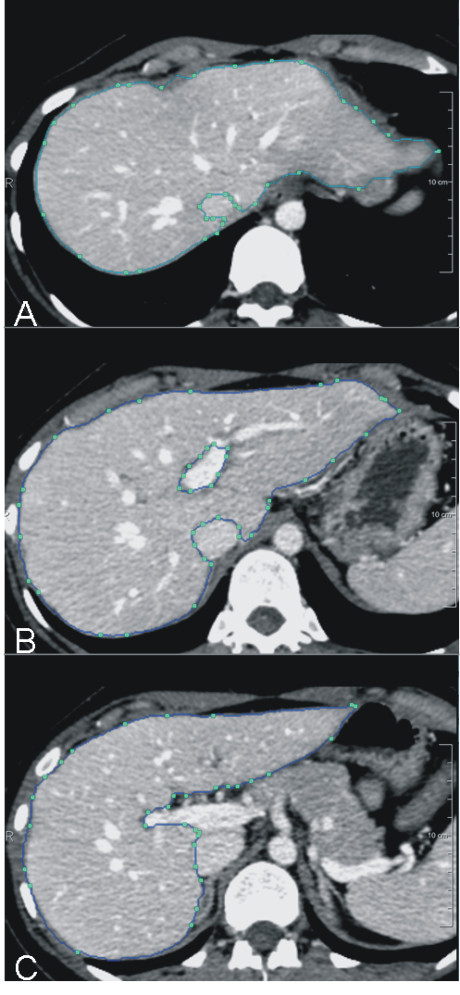
**Total liver volume was calculated using dedicated software (HepaVision®, MeVis, Germany) by tracing around the margins of the hepatic parenchyma on selected transversal slices.** Slices in between were interpolated. Large vessels as the inferior vena cava and extrahepatic portal vein were excluded. The cross sectional area (cm^2^) within the region of interest was determined, and all individual areas were summed yielding the total liver volume (cm^3^).

**Figure 2:**
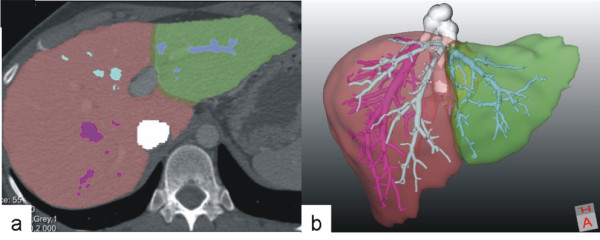
**Calculation of graft and remnant liver volume after virtual resection using HepaVision®software (MeVis, Germany).** Visualization of the resection line on transversal slices (**a**) and in 3D including hepatic veins (**b**).

Postprocessing was performed on a commercially available workstation (ADW 2.2-4.4, GE Healthcare, USA). In addition to multiplanar reformations, maximum intensity projections (MIP) and 3D volume rendered (VR) images were used for evaluation of the vascular anatomy and identifying relevant anatomic variants. Relevant steatosis was suspected when pronounced liver-spleen attenuation was observed or the attenuation of the liver parenchyma was less than the attenuation of the muscle, as suggested by previous studies [4,5]. In the relevant donors, liver biopsy was performed in order to confirm the degree of steatosis. Volume calculations of liver segments were performed using dedicated software (HepaVision®: MeVis, Germany) (Figures 1 and 2) [6,7]. Liver volumetry and segmentation was performed by an experienced radiologist, and subsequent drawing of the resection plane in agreement with the respective transplant surgeon.

## Results

**Figure 3:**
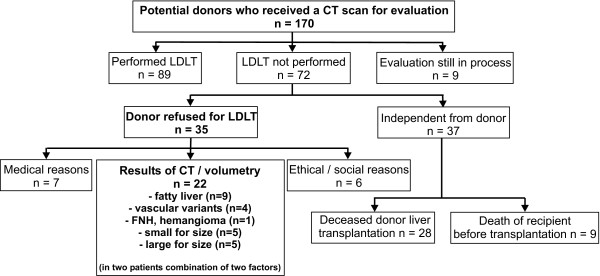
Follow-up of candidate donors being evaluated for LDLT by means of contrast enhanced CT.

**Figure 4:**
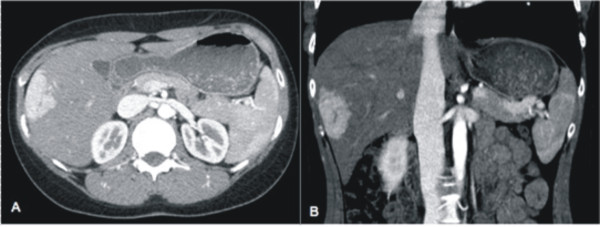
**Donor candidate in whom the left lateral segments where resected for LDLT.** Simultaneously, a FNH in the right lobe, incidentally detected in the CT scan, was resected.

After completion of the evaluation process living donor liver transplantation was realized in 89 cases, respectively 52.4% (Figure 3). These included transplantation of the left lateral liver (segments 2 and 3) in 57 patients, transplantation of a full left lobe (segments 1-4) in one patient, and transplantation of the right lobe (segments 5-8) in 31 patients (Table 1). For the right liver graft the resection line ran approximately 1 cm to the right of the middle hepatic vein. In one donor, a focal nodular hyperplasia was resected simultaneously, which was incidentally detected on the CT scan (Figure 4). In the remaining candidates surgery was not carried out either because a graft from a deceased donor became available (n = 28), death of the recipient before transplantation (n = 9) or because the donor was rejected for LDLT (n = 35). In 9 patients the evaluation process is still in progress and LDLT is planned.

**Figure 5:**
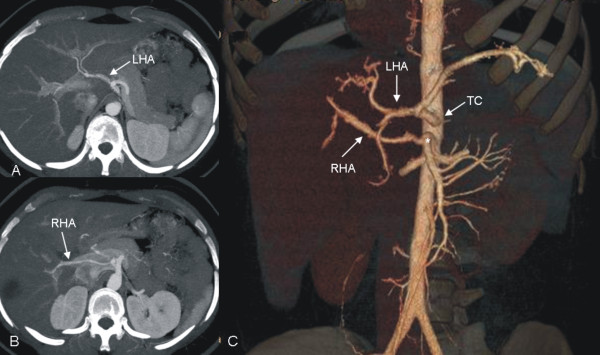
Maximum Intensity Projections (a,b) and 3D volume rendered image (c) in a candidate with a left hepatic artery (LHA) arising from the celiac trunk (TC) and a right hepatic artery (RHA) arising from the superior mesenteric artery (*). LDLT was not carried out, as graft volume was large for size.

Based on the results of contrast enhanced CT scan and liver volumetry, 22 candidates were excluded as donors. Reasons included signs of a fatty liver (n = 9) which was later on confirmed by biopsy, vascular anatomical variants (n = 4), coincidental finding of hemangioma and focal nodular hyperplasia (n = 1) or small for size (n = 5) or large for size (n = 5) graft volume, respectively. In this context small for size was defined as GRBR (graft weight to recipient to recipient body weight ratio; synonym GRWR) <0.8%. In the five potential donors calculated as large for size the relevant grafts were left lateral segments designated for children aged 5 months to 5 years with a calculated graft volume of 345 to 511 ml. The vascular variants encountered were as follows: right hepatic artery arising from the superior mesenteric artery, left hepatic artery arising from the celiac trunk in combination with a right hepatic artery arising from the superior mesenteric artery (Figure 5), and atypical venous drainage of segment 5. Candidates were further excluded in the course of the evaluation process as donors due to medical reasons, such as profound arterial hypertension and unexplained elevation of transaminases, as well as social or respectively ethical reasons (e.g. poor prognosis of the recipient).

## Discussion

To meet the need of increasing potential liver transplant recipients, alternative procedures have been developed, such as reduced-size, split and living donor liver transplantation [8]. LDLT has two major advantages over transplantation from a brain-dead donor: excellent graft quality and reduced ischemia-reperfusion injury [9].

On the other hand, donor safety is the first priority. It is important to keep in mind that LDLT should only be performed if the risk to the donor is justified by expectation of an acceptable outcome in the recipient. Overall donor morbidity is estimated to be approximately 35%, including bile leakage, wound infection and ileus [10]. A recent survey identified 33 living liver donor deaths, including 3 donors who succumbed after an attempted rescue with a liver transplant [11].

Preoperative imaging of the liver is essential in order to identify and minimize the individual risk of the potential donor. It is further determining for graft survival and in preventing vascular complications. The implementation of imaging studies of the liver, the choice of the imaging modality (CT, MRI, ultrasound, angiography, e.g.) as well as the timing of imaging (early in the evaluation process vs. in the end) varies in different transplant centers.

MRI is undoubtedly becoming more significant in the course of living donor liver evaluation, due to the development of hepatobiliary-specific contrast agents and new imaging techniques (such as chemical shift imaging, MR spectroscopy), opening up new possibilities for comprehensive imaging of the liver [12,13]. However, availability of MR is still an issue.

The purpose of this retrospective study was therefore to review the radiologist’s contribution to the process of evaluation of donors for LDLT by means of contrast enhanced CT, especially since we can look back on a large group of donors being evaluated with this technique over a significant period of time. Based on the results of the CT scan and liver volumetry in our study 22 candidates were excluded as donors, which accounts for nearly one third (31%) of the cases in which LDLT was not carried out. This relatively high number could be an argument to perform CT imaging early rather than late in the process of donor evaluation. The overall costs for complete donor evaluation have been estimated in previous studies to be in the range from 1383-2569€ [14], in some centers even as high as 4589€ [15]. Early implementation of CT imaging (estimated costs of approximately 400-600€) in the evaluation process might therefore prevent further studies in unsuitable donors and reduce costs.

Nine candidate donors were excluded because CT indicated steatosis of the liver, which was confirmed with ultrasound and liver biopsy. Fatty infiltration in hepatic grafts is known to be an important risk factor for primary graft nonfunction in deceased donor liver transplantation as well as in LDLT [16,17]. Liver grafts with a mild degree of fatty changes can be used for liver transplantation without ill effect, but liver grafts with moderate or severe degree of fatty changes have been found to have a negative effect on post transplant graft function and patient survival [17,18]. Marcos et al estimated that 1% of hepatic steatosis can decrease the functional graft mass by 1% [19]. More recently the same group published a series in which no impairment in function was found in either the living donor or the recipient using grafts containing less than 30% steatosis [20]. In our own institution the acceptable upper limit of fatty changes in the liver graft is 30%.

10 candidate donors were excluded because of the results of liver volumetry, depicting either small (n = 5) or large for size (n = 5) graft volume. Preoperative assessment of total, graft and remnant liver volume is of utmost importance, since inadequate liver mass can influence patient and graft survival. There are several formulas for calculating total liver volume depending on the body weight, body surface and gender [21,22]. Results do not always correlate and liver volume is often overestimated [23]. Depending on the individual method used, it is considered acceptable when the ratio between graft weight and recipient body weight or between graft volume and the estimated standard liver volume of the recipient are at least 0,8% and 40%, respectively [24].

In case of left lateral donation remnant liver volume is usually safe for the donor, whereas graft size might be too large in small children. In our study this led to the exclusion of five donors. Especially in case of right liver donation remnant liver volume might be too small (risking acute liver failure of the donor), whereas graft size might as well be too small resulting in a “small-for-size-syndrome” in the recipient, characterized by hepatocyte ballooning, steatosis, centrilobular necrosis and parenchymal cholestasis [25]. This led to the exclusion of three donors who were evaluated for donation of the right lobe.

CT is further important in assessment of vascular hepatic anatomy. Certain anomalies may require modification of the surgical procedure, while others might be a contraindication for surgery [26]. Other vascular variants may even be advantageous, e.g. a displaced right artery arising from the superior mesenteric artery in case of right donation. Due to the greater variability of the right hepatic vascular anatomy right hepatectomy can be one of the most challenging surgical procedures. In addition to the arterial supply of the graft and the remnant liver attention has to be paid to the venous drainage in order to prevent venous congestion. In our study 4 candidate donors were excluded from LDLT because of various vascular anatomic variants. Even though our reported vascular variants do not present absolute contraindications, in these specific cases LDLT would have been involved with an increased surgical complexity and an increased risk of graft failure respectively complications in the donor as well as in the recipient. It is important that anatomic vascular variants are assessed in context with the planned resection, and the decision whether a specific donor is suited for a specific recipient is in the end up to the responsible transplant surgeon.

## Conclusions

The use of imaging studies in the process of liver donor evaluation, specifically the choice of the imaging modality (CT, MRI, ultrasound, or angiography) as well as the timing of imaging (early in the evaluation process vs. in the end) has significant implications for the subsequent evaluation process. In our long-term experience, CT based imaging of the liver in combination with dedicated software plays a key role in the process of evaluation of candidates for LDLT. In this series almost 1/3 of donor candidates were rejected because of CT findings. CT can help to reduce the risk for donor and recipient by exclusion of unsuitable donor livers. If performed early during the evaluation process it can also prevent unnecessary studies, further reducing the risks and costs.

## Competing interests

The authors declare that they have no competing interests.

## Authors’ contributions

KIR and BR conceived and designed the experiments. KIR, CF, BPR, TB, HS and EDP performed the experiments and acquisition of data. KIR, BPR and BR analyzed and interpreted the data. TB and FW contributed materials and analysis tools. All authors participated in drafting and revising the manuscript. All authors read and approved the final manuscript.

## Pre-publication history

The pre-publication history for this paper can be accessed here:

http://www.biomedcentral.com/1471-2342/12/21/prepub

## References

[B1] Strong RW Lynch SV Ong TH Matsunami H Koido Y Balderson GA Successful liver transplantation from a living donor to her son N Engl J Med 1990 322 1505 1507 10.1056/NEJM199005243222106 2336076

[B2] Prokop M Shin H Schanz A Schaefer-Prokop CM Use of maximum intensity projections in CT angiography: a basic review Radiographics 1997 17 433 451 9084083 10.1148/radiographics.17.2.9084083

[B3] Sahani D Saini S Pena C Nichols S Prasad SR Hahn PF Halpern EF Tanabe KK Mueller PR Using multidetector CT for preoperative vascular evaluation of liver neoplasms: technique and results AJR Am J Roentgenol 2002 179 53 59 12076905 10.2214/ajr.179.1.1790053

[B4] Johnston RJ Stamm ER Lewin Hendrick RE Archer PG Diagnosis of fatty infiltration of the liver on contrast enhanced CT: limitations of liver-minus-spleen attenuation difference measurements Abdom Imaging 1998 23 409 415 10.1007/s002619900370 9663278

[B5] Panicek DM Giess CS Schwartz LH Qualitative assessment of liver for fatty infiltration on contrast-enhanced CT: is muscle a better standard of reference than spleen? J Comput Assist Tomogr 1997 21 699 705 10.1097/00004728-199709000-00004 9294555

[B6] Frericks BB Caldarone FC Nashan B Savellano D Stamm G Kirchhoff TD Shin HO Schenk A Selle D Spindler W Klempnauer J Peitgen HO Galanski M 3D CT modeling of hepatic vessel architecture and volume calculation in living donated liver transplantation Eur Radiol 2004 14 326 333 10.1007/s00330-003-2161-8 14666376

[B7] Frericks BB Kirchhoff TD Shin HO Stamm G Merkesdal S Abe T Schenk A Peitgen HD Klempnauer J Galanski M Nashan B Preoperative volume calculation of the hepatic venous draining areas with multi-detector row CT in adult living donor liver transplantation: Impact on surgical procedure Eur Radiol 2006 16 2803 2810 10.1007/s00330-006-0274-6 16710665

[B8] Alonso-Torres A Fernandez-Cuadrado J Pinilla I Parron M de Vicente E Lopez-Santamaria M Multidetector CT in the evaluation of potential living donors for liver transplantation Radiographics 2005 25 1017 1030 10.1148/rg.254045032 16009821

[B9] Kawasaki S Makuuchi M Matsunami H Hashikura Y Ikegami T Nakazawa Y Chisura H Terada M Miyagawa S Living related liver transplantation in adults Ann Surg 1998 27 269 274 9488526 10.1097/00000658-199802000-00017 PMC1191245

[B10] Barr LM Belghiti J Villamil FG Pomfret EA Sutherland DS Gruessner RW Langnas AN Delmonico FL A report of the Vancouver forum on the care of the live organ donor: lung, liver, pancreas, and intestine data and medical guidelines Transplantation 2006 81 1373 1385 10.1097/01.tp.0000216825.56841.cd 16732172

[B11] Ringe B Strong RW The dilemna of living liver donor deaths: to report or not to report? Transplantation 2008 85 790 10.1097/TP.0b013e318167345e 18360257

[B12] Ma X Holalkere NS Kambadakone RA Mino-Kenudson M Hahn PF Sahani DV Imaging-based quantification of hepatic fat: methods and clinical applications Radiographics 2009 29 1253 1277 10.1148/rg.295085186 19755595

[B13] Seale MK Catalano OA Saomo S Hahn PF Sahani DV Hepatobiliary-specific MR contrast agents: role in imaging of the liver and biliary tree Radiographics 2009 29 1725 1748 10.1148/rg.296095515 19959518

[B14] Sagmeister M Mullhaupt B Kadry Z Kullak-Unlick GA Clavien PA Renner EL Cost-effectiveness of cadaveric and living-donor liver transplantation Transplantation 2002 73 616 622 10.1097/00007890-200202270-00025 11889442

[B15] Valentin-Gamazo C Malago M Karliova M Lutz JT Frilling A Nadalin S Testa G Ruehm SG Erim Y Paul A Lang H Gerken G Broelsch CE Experience after the evaluation of 700 potential donors for living donor liver transplantation in a single center Liver Transpl 2004 10 1087 1096 10.1002/lt.20223 15349997

[B16] Todo S Demetris AJ Makowka L Teperman L Podesta L Shaver T Tzakis A Starzl TE Primary nonfunction of hepatic allografts with preexisting fatty infiltration Transplantation 1989 47 903 905 10.1097/00007890-198905000-00034 2655230 PMC2967252

[B17] D'Alessandro AM Kalayoglu M Sollinger HW Hoffmann RM Reed A Knechtle SJ Pirsch JD Hafez GR Lorentzen D Belzer FO The predictive value of donor liver biopsies for the development of primary nonfunction after orthotopic liver transplantation Transplantation 1991 51 157 163 10.1097/00007890-199101000-00024 1987685

[B18] Moon D Lee S Hwang S Kim K Ahn C Park K Ha T Song G Resolution of severe graft steatosis following dual-graft living donor liver transplantation Liver Transpl 2006 12 1156 1160 10.1002/lt.20814 16799937

[B19] Marcos A Ham JM Fisher RA Fisher RA Olzinski AT Posner MP Single-center analysis of the first 40 adult-to-adult living donor liver transplants using the right lobe Liver Transpl 2000 6 296 301 10.1053/lv.2000.6354 10827229

[B20] Marcos A Fisher RA Ham JM Shiffman ML Sanyal AJ Luketic VA Sterling RK Fulcher AS Posner MP Liver regeneration and function in donor and recipient after right lobe adult to adult living donor liver transplantation Transplantation 2000 69 1375 10.1097/00007890-200004150-00028 10798757

[B21] Urata K Kawasaki S Matsunami H Hasikura Y Ikegami T Ishizone S Momose Y Komiyama A Makuchi M Calculation of child and adult standard liver volume for liver transplantation Hepatology 1995 21 1317 1321 10.1002/hep.1840210515 7737637

[B22] DeLand FH North WA Relationship between liver size and body size Radiology 1968 91 11951 1198 10.1148/91.6.1195 5699624

[B23] Kamel IR Kruskal JB Warmbrand G Goldberg SN Pomfret EA Raptopoulos V Accuracy of volumetric measurements after virtual right hepatectomy in potential donors undergoing living adult liver transplantation Am J Roentegnol AJR 2001 176 483 487 10.2214/ajr.176.2.1760483 11159100

[B24] Chen YS Cheng YF De Villa VH De Villa VH Wang CC Lin CC Huang TL Jawan B Chen CL Evaluation of living liver donors Transplantation 2003 75 3 Suppl S16 19 12589132 10.1097/01.TP.0000046535.49186.EB

[B25] Emond JC Renz JF Ferrell LD Rosenthal P Lim RC Roberts JP Lake JR Ascher NL Functional analysis of grafts from living donors. Implications for the treatment of older recipients Ann Surg 1996 224 544 552 10.1097/00000658-199610000-00012 8857858 PMC1235420

[B26] Catalano OA Singh AH Uppot RN Hahn PF Ferrone Sahani DV Vascular and biliary variants in the liver: implications for liver surgery Radiographics 2008 28 359 378 10.1148/rg.282075099 18349445

